# New era of pulmonary embolism: Precision, intervention, and innovation

**DOI:** 10.1515/jtim-2026-0066

**Published:** 2026-08-24

**Authors:** Zhu Zhang, Haobo Li, Mengjie Duo, Zhenguo Zhai

**Affiliations:** National Center for Respiratory Medicine; State Key Laboratory of Respiratory Health and Multimorbidity; National Clinical Research Center for Respiratory Diseases; Institute of Respiratory Medicine, Chinese Academy of Medical Sciences & Peking Union Medical College; Department of Pulmonary and Critical Care Medicine, Center of Respiratory Medicine, China-Japan Friendship Hospital, Beijing, China

## Introduction

Over the past decades, pulmonary embolism (PE) remains a leading cause of morbidity and mortality worldwide. Management has shifted from broad protocols to more precise strategies. In clinical practice, physicians continue to search for validated diagnostic algorithms that support reliable decision making. Personalized treatment is increasingly replacing the traditional model in which all patients nearly receive the same care. In 2025, many high quality studies pushed the field closer to this goal. The following sections summarize the major developments in pulmonary embolism research this year (Figure 1).

**Figure 1:**
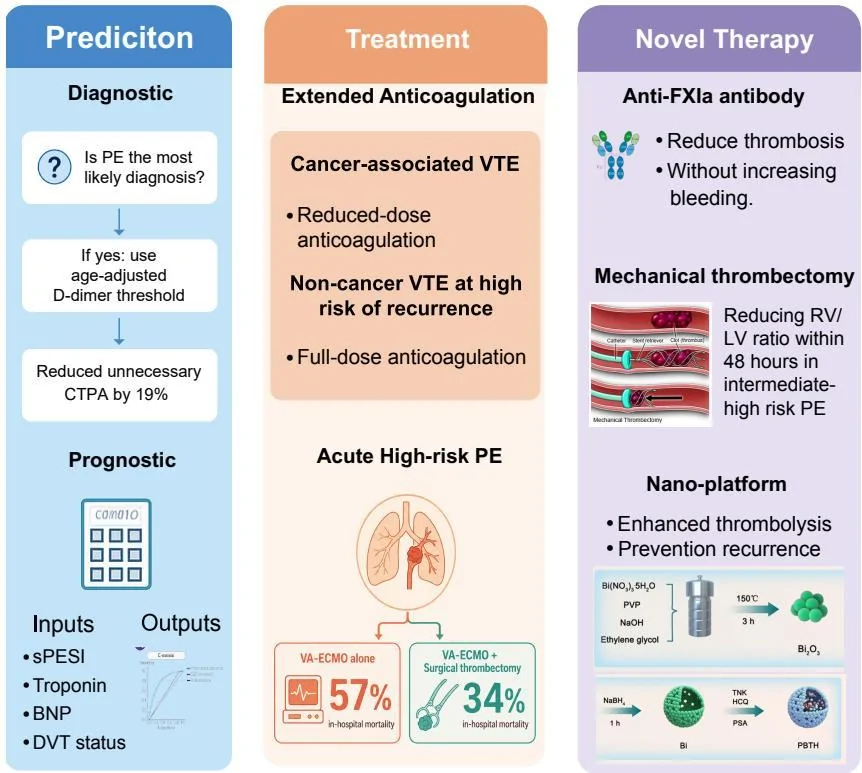
Key advances in pulmonary embolism (PE) management across prediction, treatment and novel therapies. Selected visual elements were adapted from the cited article and from publicly available resources on Drugs.com (https://www.drugs.com/cg/mechanical-thrombectomy.html).

## Streamlined diagnosis and precision prognostics

Diagnostic strategies for PE shifted towards simplification and higher precision. The modified D-dimer strategy (MODS) validated a streamlined approach where physicians raised a single question: “Is PE the most likely diagnosis?” If it is “no,” then a D-dimer below 1000 ng/mL will rule out PE without imaging, and if it is “yes,” an age-adjusted threshold should be considered. This method reduced CT pulmonary angiography (CTPA) use by 19% while maintaining a 0.00% failure rate.^[1]^ However, experts caution that the safety of this strategy relies heavily on the clinicians’ experience. Simultaneously, risk stratification has advanced from broad categories to precise calculation. The newly validated “eStiMaTe” Multimarker Calculator integrates sPESI, troponin, BNP, and DVT status. In a cohort of over 60,000 patients, it predicted 30-day mortality with superior accuracy compared to the traditional ESC model (C-statistic 0.79 *vs*. 0.56).^[2]^ Despite these advances, clinicians still face uncertainty when radiology reports are ambiguous, and many initiate anticoagulation unnecessarily to act cautiously. Furthermore, precision in biomarker assessment has also been refined. An analysis of the China pUlmonary Thromboembolism REgistry Study (CURES) involving 4105 patients highlighted the necessity of age-adjusted thresholds for natriuretic peptides to detect right ventricular dysfunction (RVD). The study established that optimal NT-proBNP cut-offs increase with age (< 55 years: 356 pg/mL; 55–69 years: 526 pg/mL; ≥ 70 years: 647 pg/mL), offering improved sensitivity for identifying high-risk patients compared to a single universal threshold. For risk assessment, a CURES registry analysis identified the RIETE score as the optimal predictor for major bleeding (AUC 0.70). This stratification proved clinically actionable, as low-risk patients treated initially with direct oral anticoagulants (DOACs) experienced significantly fewer 14-day adverse outcomes than those receiving low molecular weight heparin (HR 0.13).

## Acute management: the interventional era

The STORM-PE trial was the first RCT comparing computer assisted vacuum thrombectomy (CAVT) plus anticoagulation versus anticoagulation alone in intermediate-high risk PE. CAVT reduced the RV/LV ratio by 0.52 at 48 h, more than double the reduction in the control group, without increasing major adverse events at 7 d.^[3]^

Anticoagulation remains the cornerstone for hemodynamically stable patients, and escalation should be individualized.^[4]^ A retrospective study found that patients with lower cardiac index and marked RV dysfunction derived the greatest benefit from mechanical thrombectomy.^[5]^ Routine reperfusion for all intermediate-risk patients is not justified. Multidisciplinary evaluation should be prioritized for those with impending decompensation, heavy central clot burden, or unfavorable hemodynamic profiles.

For high-risk patients with shock or cardiac arrest, systemic thrombolysis remains the first-line reperfusion strategy in most emergency settings,^[6]^ particularly when immediate catheter-based or surgical options are not available. However, recent studies suggest that management should be further individualized in patients with profound hemodynamic collapse or failed initial reperfusion. A target trial emulation of nearly 1000 patients revealed that venoarterial extracorporeal membrane oxygenation (VA-ECMO) alone resulted in the highest in-hospital mortality of 57%, whereas combination of VA-ECMO with surgical thrombectomy was associated with the best survival outcomes, reducing mortality to 34%.^[7]^ In parallel, emerging comparative data indicate that surgical pulmonary embolectomy may provide favorable short-term outcomes compared with thrombolysis in selected patients with high-risk PE.^[8]^ Together, these findings support the view that ECMO serves best as a bridge to definitive reperfusion rather than a standalone treatment, and that reperfusion strategies for high-risk PE should be selected according to bleeding risk, treatment response, and local expertise.

## Extended anticoagulation strategies

Extended anticoagulation strategies now diverge based on cancer status. The API-CAT trial studied patients with active cancer who completed 6 months of treatment. Comparing full-dose versus reduced-dose apixaban, the reduced-dose proved non-inferior for preventing recurrence while offering superior safety by significantly lowering clinically-relevant-bleeding. This establishes dose reduction as the new standard for stable cancer patients.^[9]^

In contrast, the RENOVE trial examined non cancer patients with unprovoked VTE or persistent risk factors. Comparing full-dose direct oral versus reduced DOACs, the reduced-dose failed to meet statistical non-inferiority for recurrence risk. Consequently, non-cancer patients at high-risk of recurrence should generally maintain full-dose anticoagulation for maximum protection.^[10]^

## PE management in special populations

Obesity presents a clinical paradox where patients face higher PE risk yet often exhibit lower mortality than normal weight individuals. Diagnosis proves difficult due to imaging artifacts and reduced D-dimer specificity. Management requires dosing low molecular weight heparins (LMWH) by actual body weight and exercising caution with DOACs in cases of severe obesity.

In pregnancy, diagnostic pathways must balance maternal benefit against fetal radiation exposure. LMWH remains the preferred anticoagulant; thrombolysis or interventional reperfusion should be reserved for hemodynamic collapse or life-threatening PE.^[11]^

In older adults, PE management is complicated by frailty, renal dysfunction, polypharmacy, and elevated risks of both thrombosis and bleeding. Clinical decisions should integrate comorbidity burden, functional status, and drug interaction risk rather than chronological age alone.^[12]^

Patients at high bleeding risk represent another important subgroup in whom treatment intensity must be individualized. In these patients, the choice and duration of anticoagulation, and the decision to pursue catheter-based or surgical reperfusion, should be guided by a careful balance between the risk of hemorrhage and the expected benefit of preventing hemodynamic deterioration or recurrence. Pediatric PE displays a bimodal incidence peak in infants under one year and adolescents aged 15 to 19 years old. Mortality is extremely high in children aged 0 to 9 with high-risk features, reaching 25.3%, which underscores the need for aggressive monitoring.^[13]^ Additionally, nationwide data tracked the impact of the COVID-19 pandemic, finding a 52% incidence surge in 2021 driven by males largely. Fortunately, rates began returning to pre-pandemic levels by 2022.^[14]^

## New directions in thrombosis and anticoagulation therapy

Innovative basic science is introducing advanced therapeutic platforms. A study introduced a bismuth-based nanoplatform (PBTH) for integrated diagnosis and treatment. This “theranostic” tool targets P-selectin on activated endothelial cells and is activated by hypoxia to emit NIR-II fluorescence for precise thrombus visualization. It simultaneously releases hydrogen sulfide and drugs (TNK/HCQ) to dissolve clots and reduce inflammation.^[15]^ New anticoagulant agents targeting Factor XI are being developed to reduce thrombosis without increasing bleeding. Two phase II trials compared the Factor XI antibodies REGN9933 and REGN7508 against enoxaparin in patients undergoing knee surgery. REGN7508 proved superior to enoxaparin for preventing VTE. Importantly, neither antibody increased bleeding risk compared to enoxaparin. This confirms Factor XI as a promising target for safer anticoagulation beyond Factor Xa inhibitor.^[16]^

## Conclusion

2025 marks a new era of precision medicine for PE, driven by smarter diagnostics and advanced therapy. These developments are expected to influence future clinical guidelines and gradually reshape the standard of care.
